# Poor Sleep Health and Next Day Work Impairment: The Mediating Role of Fatigue

**DOI:** 10.1093/geroni/igaa057.1384

**Published:** 2020-12-16

**Authors:** Breann LaRocque, Christina Mu, Soomi Lee

**Affiliations:** University of South Florida, Tampa, Florida, United States

## Abstract

Nightly sleep impacts next-day alertness and cognitive functioning. For healthcare professions, work impairment can be life-threatening for patients. Thus, understanding how sleep affects work quality is imperative to promoting medical safety and overall health of workers. The current study investigated whether nightly sleep health is associated with next-day work impairment in nurses and whether this association is mediated by daily fatigue. Sixty nurses reported their sleep characteristics, fatigue, and work impairment using ecological momentary assessment for two weeks. We used a series of multilevel models (a path: sleep→fatigue, b path: fatigue→work impairment, c path: sleep→work impairment, c′ path: sleep and fatigue→work impairment), adjusting for sociodemographics and work shift. At the between-person level, poorer sleep quality was associated with greater work impairment (βc=-23.36, p<.001). This association was mediated by fatigue such that poorer sleep quality was associated with greater fatigue (βa=-19.54, p<.01), which was further associated with greater work impairment (βb=0.79, p<.001). After including fatigue, the association of sleep quality with work impairment was reduced (βc′ =-7.07, p=.08). Similarly, fatigue mediated the relationship between sleep sufficiency and work impairment (βa=-16.49; βb=0.79; βc=-19.36; p<.001; βc′ =-6.32, p=.05). At the within-person level, on days after long sleep duration (>8hrs), nurses reported greater work impairment (βc=10.08, p<.01), however, this was not mediated by fatigue. Our results suggest that poor sleep health may impair next-day work performance, mostly through increased fatigue. Future interventions for nurses can target daily fatigue to reduce the adverse effects of poor sleep on work impairment.

